# Pattern of Road Traffic Injuries and Their Pre-hospitalization Factors Reported at a Public Tertiary Healthcare Facility and Rural Private Healthcare Facility in Rajasthan, India

**DOI:** 10.7759/cureus.39390

**Published:** 2023-05-23

**Authors:** Neeraj Sharma, Vinod Kumar SV, Daya K Mangal, Yogita Sharma, Mohan Bairwa, Bontha V Babu

**Affiliations:** 1 School of Public Health, IIHMR University, Jaipur, IND; 2 Division of Socio-Behavioural, Health Systems and Implementation Research, Indian Council of Medical Research, New Delhi, IND; 3 Centre for Community Medicine, All India Institute of Medical Sciences, New Delhi, IND

**Keywords:** india, rajasthan, accident, pattern, healthcare facility, road traffic injury

## Abstract

Objective: We aimed to report the pattern of road traffic injuries (RTIs) and pre-hospitalization factors of road traffic injuries among the accident victims reported at an urban and a rural healthcare facility in the Jaipur district, Rajasthan.

Methods: This cross-sectional study was conducted in a tertiary-level, urban public healthcare facility in Jaipur city and a secondary-level, rural private facility in nearby Chomu town. The study participants were all those who encountered road traffic injury and visited any of these healthcare facilities to seek care. The study tool included information on demographics, type of road user, vehicles, accidents, roads, environment, and other pre-hospitalization factors. Data collectors were nurses trained to collect data using the tablet-based application. Data were analyzed using proportions/percentages. Bivariate analysis was done to assess the significance of differences between categories of factors and between rural and urban facilities.

Results: Among 4,642 cases, 93.8% were enrolled in the urban facility, and the remaining were enrolled in the rural facility. Predominantly, males (83.9%) and young adults 18-34 years (58.9%) were reported in both study facilities. Among the accident victims reported at the urban facility, major groups were educated up to the primary level (25.1%) or graduate level (21.9%). About 60% of them were drivers. Most of these injuries occurred on urban roads (50.2%) or two-lane roads (42%). About three-fourths of the injured were using two-wheeler geared vehicles, and 46.7% were overtaking or turning the vehicle when the accident happened. The majority of cases (61.6%) did not require hospitalization. Among the rural facility participants, 27.2% were graduates, and 24.7% were below primary education. Most of these injuries happened on the national highway (35.8%) or rural roads (33.3%). Most of them used two-wheeler geared (80.1%) at the time of the accident. Most were injured while doing normal straight driving (80.5%). Most participants (80.1%) in the rural facility did not follow the traffic rules, and 43.9% required hospitalization.

Conclusion: Young males were the most affected age group by road traffic injuries. Differential patterns of road traffic injuries and pre-hospital factors were observed in urban and rural areas.

## Introduction

Globally, most young adults aged 15-29 years die from road traffic injuries (RTIs). An estimated 50 million injuries occur each year, resulting in an estimated 1.35 million deaths [1]. Low- and middle-income countries (LMICs) account for about 85% of the total deaths and 90% of disability-adjusted life years (DALYs) lost due to RTIs [2], and India is no exception. A total of 449,002 road accidents were reported in India in 2019, causing injuries to 451,361 people and claiming 151,113 lives [3].

The death rate and injury rate (11.54 and 33.74 per 100,000 population in 2018, respectively) remained almost static in India since 2015 (11.81 and 38.31 per 100,000 population, respectively) [4]. Similarly, in Rajasthan, a large state in India, the total number of cases of RTI increased from 21,743 in 2018 to 23,480 in 2019 [3]. Most of these untoward incidences of RTI (>80%) happened due to the neglect of some pre-identified preventable factors [5].

These factors comprise human factors such as behavioral/social factors (drinking and driving, speeding, and violating traffic laws) and impaired skills (lack of attention, exhaustion, and physical disabilities), vehicular factors (poor vehicle light, inefficient braking system, and lack of safety measures), infrastructural factors (poor road conditions, insufficient safety, and absence of signage on the road), and environmental factors (poor weather and light condition) [5-7]. Other critical pre-hospitalization factors are the type of accident, pre-hospitalization intervals, first aid facilities availed, transportation facilities, and nature of injury (single-organ or multi-organ injuries, and blunt or penetrating injuries) [8,9]. These factors affect ideal driving situations in various permutations and combinations and may lead to fatal and non-fatal accidents.

Good quality evidence on the dynamics of RTIs is the key to developing potential solutions and their effective implementation. Developed countries conducted ample research on RTIs and related factors and effectively implemented their interventions to reduce the burden of RTIs [10]. On the other side, little knowledge exists on the factors contributing to the severity of road accidents and the usage of effective prevention strategies in LMICs [11-13].

India is a diversified country where each state presents different geographical and population characteristics that indirectly enforce state-specific research agendas. Simultaneously, India faces a rapid increase in motor vehicles including all types of vehicles, with six million new vehicles sold yearly, which is expected to surpass the number of cars on its roads by 2050 [14]. Rajasthan is a state with poor health indicators, where maternal and child health indicators are improving, but the share of RTIs in the state is either increasing or stable apart from the COVID-19 pandemic years [13]. Existing evidence primarily focused on the demographic and socioeconomic characteristics, and clinical features, unintentionally presenting a curtailed image of the RTI situation in the state [13,15-17]. This incomplete information directly or indirectly affects the evidence-based decision-making process. To fill such a gap, we conducted this analysis to explore the pattern of RTIs and pre-hospitalization factors among the injured reported at a tertiary-level urban public healthcare facility and a secondary-level rural private healthcare facility in Rajasthan.

## Materials and methods

Study setting

This study was part of a pan-India multicentric study on the “Establishment of a Comprehensive Surveillance System for Road Traffic Injuries in India” (herewith known as the “IRIS study”). Any fatal or non-fatal injury incurred as a result of a collision on a public road involving at least one moving vehicle was included in this study. A detailed IRIS study protocol is available elsewhere [18].

This study was cross-sectional in nature. The study was conducted in a tertiary-level public healthcare facility situated in urban areas (here onward referred to as an “urban facility”) and a secondary-level private healthcare facility in rural areas (here onward referred to as a “rural facility”). The urban facility is located in Jaipur city, while the rural facility is situated around 30 km from Jaipur in Chomu town on a national highway.

Study participants and study duration

Both facilities had a designated trauma unit to manage and treat RTI cases. All RTI cases who visited to avail of healthcare services from the selected facilities were enrolled in this study. We used a tablet-based, structured interview schedule for this study. The data was collected from November 2017 to May 2018 (hereafter, it will be known as IRIS data).

Participant’s enrollment process and data collection

A team of research investigators was recruited. The investigators were nurses having two-year diploma courses as prescribed by the government of India. They were provided with three days of training, followed by a half-day refresher every three months, to collect data for the study. The investigators were available at the study facilities around the clock. The investigators explained the aim of the study to the victims of RTIs when they reported to the study facilities. The data was captured in a tablet-based study tool developed for the IRIS study. The respondents were mainly injured. If the victims cannot be interviewed, their attendants or relatives have provided the relevant information during hospitalization. The investigators also accessed hospital records of RTI cases to check for missing data. Other information on demographics, type of road user, vehicles, accidents, roads, environment, and other pre-hospitalization factors was also collected.

Data flow and data management

The study tool was developed, tested, and implemented on an electronic platform. The platform allowed study team members to gather data electronically across multiple tablets and sites and store it in the cloud. Without any individual identifier, this data was stored on a password-protected laptop. The data were cleaned using Excel (Microsoft Corp., Redmond, WA, USA) and R software to identify the implausible information during data cleaning and rectified by revisiting the responses in the original datasheet. Cases with incomplete outcome information were dropped (n = 15). Based on the initial review, we found that the final data set would be sufficient for further analysis.

Data analysis

After data cleaning, statistical analysis was performed using R software (version 4.4.1.0) and R studio (version 1.4.1717). All variables were categorical, so we presented the data in percentages or proportions. The tables of descriptive analysis were subcategorized according to the healthcare facilities.

Bivariate analysis was performed for the following pre-hospitalization factors: sociodemographic factors such as age, gender, education level, occupation, and type of road user; road characteristics such as the type of road, a subtype of road, and road surface condition; vehicle characteristics such as the type of vehicle involved and vehicle maneuver; environmental characteristics such as weather condition, light condition, and the date and time of the accident; pre-hospital characteristics and hospitalization such as traffic rules followed, time for rescue, rescued by, first aid given, if the patient reached in the golden hour, and the outcome of RTI as hospitalization; and accident characteristics such as accident type, injured status at the time of admission, and the number of body parts injured. The chi-square test or Fisher’s exact test was used to find the significance of the difference between rural and urban health facilities. The significance level was set at 0.05.

## Results

In this study, 4,642 cases of road traffic injuries (RTIs) were reported, including 4,354 (93.8%) in the urban facility and 288 (6.2%) in the rural facility. Predominantly, males were reported at both study sites (urban facility: 84.2%, rural facility: 78.8%). The most affected participants were those who belonged to the 18-34 age group (urban facility: 59.3%; rural facility: 55.1%). One-fourth of study participants reported in the urban facility had primary education, and 21.9% had graduate or above education. About one-fourth of study participants in the rural health facility were educated below the primary level, and 27.2% were educated as graduates or above.

In the urban facility, unskilled workers were the predominant group reported (19.9%), followed by service employees (16.7%), homemakers (10.5%), and students (5.9%). Homemakers were the most predominant group (15.6%) in the rural facility, followed by service employees (14.5%), unskilled workers (12%), and agricultural workers (11.7%). Most of the victims were drivers (urban facility: 60.5%, rural facility: 55.9%). Others were passengers, pedestrians, and pillion riders involved in much lower proportions, ranging from 11% to 14%. More pedestrians were reported in the urban facility (14.4%) compared to the rural facility (6.3%). A higher proportion of injured pillion riders were reported in the rural facility than in the urban facility (19.1% versus 11.1%) (Table 1).

**Table 1:** Sociodemographic characteristics of study participants

Variable	Subcategory	Urban facility (number (%))	Rural facility (number (%))	Total (number (%))	Significance (p-value)
Gender	Female	687 (15.8)	61 (21.2)	748 (16.1)	0.020
	Male	3,667 (84.2)	227 (78.8)	3,894 (83.9)	
	Total	4,354 (100)	288 (100)	4,642 (100)	
Age group (years)	Below 18	75 (1.7)	7 (2.5)	82 (1.8)	0.212
	18-24	1,296 (29.8)	65 (22.8)	1,361 (29.3)	
	25-34	1,283 (29.5)	92 (32.3)	1,375 (29.6)	
	35-44	750 (17.2)	50 (17.5)	800 (17.2)	
	45-59	647 (14.9)	49 (17.2)	696 (15)	
	60 and above	306 (7)	22 (7.7)	328 (7.1)	
	Total	4,357 (100)	285 (100)	4,642 (100)	
Education status	Below primary	732 (17)	71 (24.7)	803 (17.4)	<0.001
	Primary	1,085 (25.1)	36 (12.5)	1,121 (24.4)	
	High school	827 (19.2)	55 (19.2)	882 (19.2)	
	Higher secondary	724 (16.8)	47 (16.4)	771 (16.8)	
	Graduate or above	947 (21.9)	78 (27.2)	1,025 (22.3)	
	Total	4,315 (100)	287 (100)	4,602 (100)	
Occupation	Homemaker	456 (10.6)	44 (15.5)	500 (11)	<0.001
	Professional/business	31 (0.7)	0 (0)	31 (0.7)	
	Self-employed	238 (5.5)	18 (6.4)	256 (5.6)	
	Agriculture	203 (4.7)	33 (11.7)	236 (5.1)	
	Government/private employee	717 (16.7)	41 (14.5)	758 (16.5)	
	Skilled manual laborer	463 (10.8)	8 (2.8)	471 (10.3)	
	Unskilled manual laborer	858 (19.9)	34 (12)	892 (19.5)	
	Unemployed	146 (3.4)	0 (0)	146 (3.2)	
	Student	257 (6)	11 (3.9)	268 (5.8)	
	Others	932 (21.7)	94 (33.2)	1,026 (22.4)	
	Total	4,301 (100)	283 (100)	4,584 (100)	
Type of road user	Driver	2,619 (60.5)	161 (55.9)	2,780 (60.2)	<0.001
	Passenger	606 (14)	54 (18.8)	660 (14.3)	
	Pedestrian	623 (14.4)	18 (6.3)	641 (13.9)	
	Pillion rider	482 (11.1)	55 (19.1)	537 (11.6)	
	Total	4,330 (100)	288 (100)	4,618 (100)	

Road characteristics

Most RTI victims who reported at the rural facility had an accident either on the national highway (35.8%) or on rural roads (33.3%). Among the urban facility participants, more than half of the accident victims had an accident on urban roads. A contrasting pattern of injuries was reported at urban and rural facilities regarding the road lanes. The highest proportion of accidents occurred on four-lane roads (38.2%) in the rural facility, followed by two-lane roads (35.7%), while these proportions reversed in the urban facility (30.1% versus 42%) (Table 2).

**Table 2:** Distribution of road traffic injuries by road characteristics

Variable	Subcategory	Urban facility (number (%))	Rural facility (number (%))	Total (number (%))	Significance (p-value)
Type of road	National highway	787 (18.2)	103 (35.8)	890 (19.2)	<0.001
	State highway	576 (13.3)	49 (17)	625 (13.5)	
	Urban road	2,176 (50.2)	40 (13.9)	2,216 (47.9)	
	Rural road	798 (18.4)	96 (33.3)	894 (19.3)	
	Total	4,337 (100)	288 (100)	4,625 (100)	
Subtype of road	One lane	1,124 (27.9)	74 (26.1)	1,198 (27.8)	0.015
	Two lane	1,690 (42)	101 (35.7)	1,791 (41.6)	
	Four lane	1,213 (30.1)	108 (38.2)	1,321 (30.7)	
	Total	4,027 (100)	283 (100)	4,310 (100)	
Road surface condition	Dry	4,039 (93.1)	266 (92.4)	4,305 (93)	0.209
	Rutted/pot-holed	210 (4.8)	19 (6.6)	229 (5)	
	Others	90 (2.1)	3 (1)	93 (2)	
	Total	4,339 (100)	288 (100)	4,627 (100)	

Vehicle characteristics

Most victims reported using two-wheeler geared vehicles (urban facility: 74.3%, rural facility: 80.1%), followed by ≥3-wheeler commercial vehicles (14.1% versus 18.1%) while accidents happened. The proportion of participants using non-geared two-wheelers was much lower in the rural facility than in the urban facility (1.4% versus 7.2%). Most participants in the rural facility reported straight driving at the time of the accident (80.5%), while in the urban facility, overtaking or turning (46.7%) was the predominant maneuver, followed by straight driving (36.3%) (Table 3).

**Table 3:** Distribution of road traffic injuries by vehicle-related characteristics

Variable	Subcategory	Urban facility (number (%))	Rural facility (number (%))	Total (number (%))	Significance (p-value)
Type of vehicle	Two-wheeler (geared)	3,169 (74.3)	230 (80.1)	3,399 (74.7)	<0.001
	Two-wheeler (non-geared)	305 (7.1)	4 (1.4)	309 (6.8)	
	Four-wheeler (personal)	105 (2.5)	1 (0.4)	106 (2.3)	
	≥3-wheeler (commercial)	603 (14.1)	52 (18.1)	655 (14.4)	
	Others	84 (2)	0 (0)	84 (1.8)	
	Total	4,266 (100)	287 (100)	4,553 (100)	
Vehicle maneuver	Normal straight driving	1,531 (36.3)	231 (80.5)	1,762 (39.1)	<0.001
	Overtaking	1,088 (25.8)	10 (3.5)	1,098 (24.4)	
	Turning	880 (20.9)	23 (8)	903 (20)	
	Slowing/stopping/moving off	609 (14.4)	22 (7.7)	631 (14)	
	Others	113 (2.7)	1 (0.3)	114 (2.5)	
	Total	4,221 (100)	287 (100)	4,508 (100)	

Environmental characteristics

Almost all accident victims reported having an accident during the clear weather, and the majority of participants met an accident during daylight (rural facility: 76%, urban facility: 64.4%, p < 0.05). About 39% of cases had an injury between 15.00 and 20.59 hours. In the rural facility, the proportion of victims reported on the weekend was relatively higher (19%-22%). At the same time, it was almost equally distributed (12%-16%) in the urban facility (Table 4).

**Table 4:** Distribution of road traffic injuries by environmental characteristics

Variable	Subcategory	Urban facility (number (%))	Rural facility (number (%))	Total (number (%))	Significance (p-value)
Weather conditions	Clear	4,319 (99.2)	287 (99.7)	4,606 (99.2)	0.611
	Not clear	35 (0.8)	1 (0.3)	36 (0.8)	
	Total	4,354 (100)	288 (100)	4,642 (100)	
Light conditions	Daylight	2,803 (64.4)	219 (76.1)	3,022 (65.1)	<0.001
	Semidarkness	404 (9.3)	3 (1)	407 (8.8)	
	Darkness	1,145 (26.3)	66 (22.9)	1,211 (26.1)	
	Total	4,352 (100)	288 (100)	4,640 (100)	
Time	0.00-2.59	148 (3.4)	18 (6.3)	166 (3.6)	0.013
	3.00-5.59	87 (2)	3 (1)	90 (1.9)	
	6.00-8.59	428 (9.8)	37 (12.9)	465 (10)	
	9.00-11.59	730 (16.8)	36 (12.5)	766 (16.5)	
	12.00-14.59	725 (16.7)	52 (18.1)	777 (16.8)	
	15.00-17.59	826 (19)	63 (21.9)	889 (19.2)	
	18.00-20.59	883 (20.3)	44 (15.3)	927 (20)	
	21.00-23.59	525 (12.1)	35 (12.2)	560 (12.1)	
	Total	4,352 (100)	288 (100)	4,640 (100)	
Day	Monday	580 (13.3)	35 (12.2)	615 (13.3)	0.003
	Tuesday	556 (12.7)	31 (10.8)	587 (12.7)	
	Wednesday	667 (15.3)	34 (11.8)	701 (15.1)	
	Thursday	576 (13.2)	42 (14.6)	618 (13.3)	
	Friday	654 (15)	27 (9.4)	681 (14.7)	
	Saturday	632 (14.5)	55 (19.1)	687 (14.8)	
	Sunday	689 (15.8)	64 (22.2)	753 (16.2)	
	Total	4,354 (100)	288 (100)	4,642 (100)	

Pre-hospital characteristics and hospitalization

Among the urban facility participants, almost 50% reported following the traffic rules at the time of an accident, whereas only 20% were following the traffic rules among the rural facility participants. All injured victims reported at the rural facility received help within half an hour at the accident site, whereas 93.1% of their urban counterparts were rescued within half an hour. Local people or passersby rescued most participants (urban facility: 60%, rural facility: 71%). In 11% of urban facility participants and 3.9% of rural counterparts, self-rescue was done, whereas police rescued 3.9% of urban facility participants. Among the participants, only 20.2% received first aid near the accident site before reaching the health facility. About 19% of the urban facility participants reported that they could get to a nearby hospital within the golden hour for emergency treatment. Surprisingly, this proportion is higher in the rural facility (33.3%). It was found that four out of 10 participants needed hospitalization (urban facility: 38.4%, rural facility: 43.9%) (Table 5). Victims who died at the accident site, on the way, or during the data collection period were included in the outcome as hospitalized.

**Table 5:** Distribution of road traffic injuries by pre-hospital characteristics and hospitalization

Variable	Subcategory	Urban facility (number (%))	Rural facility (number (%))	Total (number (%))	Significance (p-value)
Following traffic rules while accident happened	Yes	1,873 (49.7)	54 (19.9)	1,927 (47.7)	<0.000
	No	1,898 (50.3)	217 (80.1)	2,115 (52.3)	
	Total	3,771 (100)	271 (100)	4,042 (100)	
Time for rescue	Within half an hour	4,002 (93.1)	287 (100)	4,289 (93.5)	<0.000
	Within half an hour to an hour	168 (3.9)	0 (0)	168 (3.7)	
	More than an hour	128 (3)	0 (0)	128 (2.8)	
	Total	4,298 (100)	287 (100)	4,585 (100)	
Rescued by	Self	474 (11)	11 (3.9)	485 (10.5)	<0.000
	Police	168 (3.9)	0 (0)	168 (3.7)	
	Local people	2,592 (60.1)	203 (71)	2,795 (60.7)	
	Relatives	1,021 (23.7)	64 (22.4)	1,085 (23.6)	
	Driver/passenger	61 (1.4)	8 (2.8)	69 (1.5)	
	Total	4,316 (100)	286 (100)	4,602 (100)	
First aid given	Yes	860 (20.34)	46 (18.25)	906 (20.22)	0.471
	No	3,368 (79.66)	206 (81.75)	3,574 (79.78)	
	Total	4,228 (100)	252 (100)	4,480 (100)	
First aid given site	At the accident site	213 (24.77)	4 (8.70)	217 (23.95)	0.003
	Nearby government hospital	371 (43.14)	34 (73.91)	405 (44.70)	
	Nearby private clinic	222 (25.81)	7 (15.22)	229 (25.28)	
	Ambulance	49 (5.70)	1 (2.17)	50 (5.52)	
	Others	5 (0.58)	0 (0)	5 (0.55)	
	Total	860 (100)	46 (100)	906 (100)	
Patient reached in golden hour	Yes	822 (18.9)	96 (33.3)	918 (19.8)	<0.000
	No	3,532 (81.1)	192 (66.7)	3,724 (80.2)	
	Total	4,354 (100)	288 (100)	4,642 (100)	
Hospitalization made	No	2,509 (61.6)	156 (56.1)	2,665 (61.2)	0.002
	Yes	1,567 (38.4)	122 (43.9)	1,689 (38.8)	
	Total	4,076 (100)	278 (100)	4354 (100)	

Accident characteristics

The pattern of the accident types, such as vehicle-to-vehicle collision (40%) and self-fall/skid (33%), was comparable in both facilities. Vehicle-to-pedestrian accidents were more common among those reported in the urban facility (14.8% versus 6.3%), and vehicle-to-animal or moving object accidents were higher in the rural facility (10.8% versus 5.9%). Most study participants (90.3%) were conscious during admission, followed by 8.4% who were unconscious, and 1.3% brought in dead. About three-fourths of the study participants in the rural facility had more than two body parts injured in the accident, compared with 55% in the urban facility (Table 6). We also compared the participants’ characteristics of those whose outcomes were unavailable. These participants also reported similar characteristics.

**Table 6:** Distribution of road traffic injuries by accident characteristics

Variable	Subcategory	Urban facility (number (%))	Rural facility (number (%))	Total (number (%))	Significance (p-value)
Accident type	Self-fall/skid	1,432 (33)	95 (33)	1,527 (33)	<0.001
	Vehicle to vehicle	1,760 (40.5)	116 (40.3)	1,876 (40.5)	
	Vehicle to pedestrian	642 (14.8)	18 (6.3)	660 (14.2)	
	Vehicle to animal/moving object	254 (5.8)	31 (10.8)	285 (6.2)	
	Others	257 (5.9)	28 (9.7)	285 (6.2)	
	Total	4,345 (100)	288 (100)	4,633 (100)	
Injured status at the time of admission	Unconscious	359 (8.3)	27 (9.4)	386 (8.4)	0.266
	Conscious	3,914 (90.3)	260 (90.3)	4,174 (90.3)	
	Brought dead	61 (1.4)	1 (0.4)	62 (1.3)	
	Total	4,334 (100)	288 (100)	4,622 (100)	
Number of body parts injured	One	773 (17.8)	20 (6.9)	793 (17.2)	<0.001
	Two	1,173 (27)	50 (17.4)	1,223 (26.4)	
	Three	1,226 (28.3)	106 (36.8)	1,332 (28.8)	
	Four or more	1,165 (26.9)	112 (38.9)	1,277 (27.6)	
	Total	4,337 (100)	288 (100)	4,625 (100)	

## Discussion

We aimed to study the pattern of RTIs and pre-hospitalization factors of RTIs among two healthcare facilities, one each from a rural and urban area. Most of the injured persons reported at these facilities were male. The overall age distribution was similar among both facility participants. The highest proportion of study participants belonged to the 18-34 years age group. Studies from other parts of the country, i.e., Hyderabad [19], Maharashtra [20], and Jaipur [21], also reported similar findings, ranging between 55% and 60%. This age group also contributes to the highest mortality and disability due to road traffic injuries in India [15]. Years of lives lost due to premature deaths further impact national productivity and economic growth. Hence, the existing burden is a cause for concern in India.

Most urban facility participants had accidents on urban roads (50.2%) or two-lane roads (42%). The rural facility was situated on the national highway to connect two major cities (Jaipur and Ajmer) of Rajasthan state; hence, a large proportion of participants had an accident on the national highway (35.8%) or the four-lane roads (38.2%). Among the total reported participants at the urban facility, a significantly higher proportion were pedestrians (14.4%) compared to the rural facility (6.3%). There are no proper footpaths for pedestrians to walk on in the city, where pedestrians are at a higher risk of accidents. While a lot of efforts have been made to protect vehicle occupants, the needs of pedestrians were not met in India [22]. Urban healthcare facility situated in the heart of the city; hence, it is easily accessible to accident victims. A similar pedestrian proportion was reported among the findings from previous studies in urban settings [20,23]. It could be because footpaths are encroached on by the traders, which forces pedestrians to walk on busy roads and leads to accidents [24]. This is evident from Operation Pink, the government of Rajasthan’s initiative in Jaipur to remove the encroachment in the year 2000. After over 2,271 written complaints in a year, the initiative was revived in 2012, 2019, and 2021 [25].

Most study participants in rural areas used two-wheeler geared vehicles (80.1%) when the accident happened, and they performed normal straight driving (80.5%). These findings were similar to the previous studies, where most accident victims were two-wheeler occupants [24,26]. Additionally, in recent years, registration of non-geared two-wheeler vehicles has increased [27,28], and it was understandable to see some proportion (7.2%) of cases using these vehicles in the urban area at the time of an accident. Previous studies also reported the vulnerability of pedestrians and two-wheelers to road traffic accidents and fatal injuries [29,30].

The proportion of RTIs from the rural facility showed an upward trend from morning to afternoon. Interestingly, the proportion showed a decreasing trend that tapered off until early morning, 3.00-5.59 (1%). In the urban facility, the accidents showed a slower rise with a lower proportion in the morning at 6.00-8.59 (9.8%), although the slow but rising trend continues to reach the peak between 18.00 and 20.59 (20.3%) and then decrease till the early morning. This pattern depicts the lifestyle differences between rural and urban settings. In rural areas, people get up early, start work early, and return home early in the evening. In contrast, people residing in urban areas and working in localities usually start late and work until late hours.

People tend to follow traffic rules more in urban areas than in rural areas [31]. However, the excess number of accidents in urban areas is due to the higher density of people and vehicles [32]. About half of the study participants, in our study, in the urban facility reported following traffic rules at the time of the accident. In contrast, only one-fifth of the cases at the rural facility reported following the traffic rules at the time of the accident. The findings were similar to a previous study from Rajasthan in that participants from urban settings tend to follow more traffic rules than the rural setting participants (70.5% in urban versus 8.6% in rural) [31]. The rural facility is on a national highway outside the city, so people may be less aware of following the traffic rules [33]. Law enforcement agencies should design awareness campaigns focusing on the young population of rural and semi-urban areas close to national highways in collaboration with educational institutions. Previous studies have reported that educational programs may help increase knowledge and influence the behavior of drivers and pedestrians but have little impact on reducing crash rates. Hence, such measures should be supplemented with new legislation and/or strict law enforcement [23].

Three-fourths of the participants in the rural facility had more than two body parts injured, and 43.9% required hospitalization, compared to a lesser proportion of the participants in the urban facility who had more than two body parts injured (55.1%) and required hospitalization (38.4%). Given their poor traffic behavior, this indicates the probability of severe highway accidents and the vulnerability of the people living in rural localities. It might be due to a speeding factor or disobeying traffic laws. It may require further studies to establish a causal relationship [6].

This study has also demonstrated that a reasonably good surveillance system may be implemented with the help of nurses and an electronic-based system in both rural and urban areas. This study provided a good account of patterns of RTIs and pre-hospitalization factors in both rural and urban areas. Such evidence not only can help local law enforcement agencies for improving traffic awareness among citizens but also prepares the ground for prospective follow-up studies to explore the outcomes and plan intervention studies to assess the impact of interventions on RTI patterns.

Limitations

There were a few limitations in the study. The sample from urban and rural was not proportional. The selected urban facility is a well-equipped, tertiary-level, and the largest public healthcare facility in the city. It has round-the-clock services for trauma patients. The rural facility is also a major trauma care facility with surgery, orthopedics, neurosurgery, and critical care services in the concerned catchment area. Large trauma care facilities are mainly located in urban areas only; hence, the RTI pattern presented in the urban facility does not necessarily represent the RTIs that occurred in urban areas, but these could include the RTIs that occurred in rural areas too. However, a substantial proportion of RTIs used to happen in urban areas. There is scarce evidence available on the patterns of RTIs and relevant factors in both urban and rural healthcare facilities for preventive measures. Hence, this study will help fill the gap to some extent.

The study findings are based on the information provided by the study participant or their closest relative present in the hospital on the first day an injured person reported to the hospital. The study team collected necessary clinical data from hospital records, which are already included in the analysis. The information collected was not in real time but was captured at the earliest possible time after the accident.

## Conclusions

The study provided an account of RTIs reported to urban and rural health facilities in the Jaipur district of Rajasthan. We found that young males were the most affected by RTIs in both urban and rural areas. About half of the participants did not follow traffic rules, only 20% received first aid, and a similar proportion could reach the facility within the golden hour. The findings of the study can be helpful for local law enforcement authorities to take preventive measures for RTIs.
